# Diarrheal disease in under-five children among model and non-model families in northern Ethiopia, 2017: a comparative cross-sectional study

**DOI:** 10.1186/s13104-019-4322-0

**Published:** 2019-05-28

**Authors:** Berhe Beyene Gebrezgiabher, Teklehaymanot Huluf Abraha, Desalegn Tetemke, Gebreamlak Gidey, Negasi Asres, Alias Tesfamariam, Zemichael Weldegebrial, Teklit Angesom, Mebrahtu Abay

**Affiliations:** 1 Department of Epidemiology and Biostatistics, College of Health Sciences, Aksum University, P.O. Box: 298, Aksum, Ethiopia; 2 Department of Reproductive Health, Health Science College, Aksum University, P.O. Box: 298, Aksum, Ethiopia; 3 Department of General Public Health, College of Health Sciences, Aksum University, P.O. Box: 298, Aksum, Ethiopia; 4 Department of Midwifery, College of Health Sciences, Aksum University, P.O. Box: 298, Aksum, Ethiopia; 5 Department of Biomedical Sciences, College of Health Sciences, Aksum University, P.O. Box: 298, Aksum, Ethiopia

**Keywords:** Diarrhea, Model and non-model families, Ethiopia

## Abstract

**Objective:**

Diarrheal disease in under-five children among model families is expected to be lower than non-model families. Therefore, this study compared the prevalence and associated factors of diarrheal diseases among under-five children between model and non-model families. A comparative cross-sectional study was conducted from May to June 2017 among 322 children from each model and non-model family. Using multistage sampling technique data were collected through interview and observation. Both bi-variable and multivariable analyses were used to compute the statistical associations. Statistical significances were declared at 95% CI and *p* value < 0.05.

**Results:**

Diarrheal disease in under-five children for those from model families was 26 (8.1%) and 65 (20.2%) to the non-model families with 95% CI 0.117, 0.168. Being non-model family (AOR = 1.9 and 95% CI 1.004, 3.565), maternal history of diarrhea (AOR = 3.3 and 95% CI 1.975, 5.570), improper waste disposal method (AOR = 2.6 and 95% CI 1.251, 5.578) and not latrine use (AOR = 2.1 and 95% CI 1.128, 3.897) were found determinant factors of diarrhea. Health extension model families training and follow up programs are needed to be expanded for all non-model families.

## Introduction

According to different reviews conducted in Ethiopia, basic health services in the rural areas of the country are still with limited access. To fill this gap, the Ethiopian government is currently implementing an interventional package, the so-called health extension package (HEP), which was first introduced in the country by 2003 [1, 2]. The program delivers a package of activities which aimed at promoting, preventing and curing health problems of the community at household (HH) level [3].

The HEP consists of 16 elements. These are tuberculosis, HIV, malaria, maternal and child health, family planning, immunization, adolescent reproductive health, nutrition, excreta, waste disposal, water supply, food hygiene, housing, personal hygiene, vector and rodent control and health education and communication [4]. Implementation of the HEP is through continuous education and training of the households in the community to fully practice the packages.

The health extension workers identify and train “model families” that have acceptance and credibility by the community and are early adopters of desirable health practices. The model families, as role models, then help practically disseminate health messages which leads the “non-model families” to easily adopt desire health practices and behaviors [3].

Mortality of under-5 years old children is often used as an indicator of health status of the society [5]. Among the health problems of under-five children in low-income countries, diarrheal disease contributes a significant proportion [6]. Diarrhea in under-five children is defined as a child with loose of watery stool for three or more times during a 24-hour’s period. In underdeveloped and developing countries diarrheal diseases are still the main public health problem of children aged less than 5 years [6]. It is a leading cause of preventable death, especially among under-five children in developing countries [7]. Globally, diarrheal disease is the second most common cause of death in under-5 years old children [8]. Each year approximately two million people, the majority of whom are under-five children, die from diarrheal disease in developing countries [9].

The 2 weeks prevalence of diarrheal disease in Ethiopia ranges from 10 up to 40% [10]. According to the Ethiopian Demographic and Health Survey (EDHS, 2016) report, 12% of children in Ethiopia ever had diarrhea within 2 weeks preceding the survey [5]. The occurrence and severity of diarrhea is accelerated by lack of access to adequate clean water, unhygienic disposal of human wastes, improper solid and liquid waste disposal, poor housing conditions and lack of access to adequate and affordable health care services [5].

Diarrheal disease still continues to be a rampant public health problem among under-5 years old children [11, 12]. Though the Ethiopian government has made efforts in expanding health extension program for the communities to have improved health outcome, there is still limited evidence on the effect of health extension program on prevention and control of diarrheal disease. Therefore, this study aimed at comparing the level of diarrheal disease and associated factors among under-five children between health extension model and non-model families.

## Main text

### Methods

#### Study design and setting

A community based comparative cross-sectional study design was carried out from May to June 2017 in L/Maychew district, Tigray, Ethiopia. L/Maychew district is located in northern Ethiopia 1067 km away from Addis Ababa which is the capital city of Ethiopia. The study population for health extension model families were households with under-five children trained, graduated and certified for health extension package completion by the district health office, whereas study population for non-model families were households with under-five children who were trained or not, not graduated and certified by the district health office. HHs that successfully implement and use all the components of HEP and certified by district health office were considered as model families.

#### Sample size and sampling procedure

Sample size was calculated by Epi-Info version-7 software by assuming; proportion of model families 9.3%, proportion of non-model families 14.1%, odds ratio 2.65 [4], 5% level of significance, 95% confidence level 80% power of the study, 1:1 model to non-model ratio, 5% non-response and two design effect. Accordingly, 326 under-five children from model and non-model families each were included for the study. A multistage sampling technique was applied and sampling was done at Kebele (small administrative unit in the district) and HH levels. Under-5 year children residing in health extension model and non-model families were identified through a house to house enumeration prior to the actual data collection. Proportional sample allocation was done for each Kebeles. In the first stage, six out of 16 Kebeles were randomly selected. From these Kebeles, households with children under-5 years were selected using systematic random sampling technique. Family members of less than 18 years old were excluded from the interview by considering that correct data obtaining is difficult from those individuals. Finally, after obtaining written consent, data were collected until the sample size makes up 326 from health extension model and non-models families each.

#### Data collection and analysis

Data was collected using interviews and observation by eight diploma-level nurses supervised by four bachelor health professionals. Two-day training on the objective of the study, confidentiality of information and way of collecting data were given. A way of disposal refuses, which includes burning, buried in pit or store in a container, compost, and disposed of in the designed site were considered as proper waste disposal. Pre-coding and manual checking of the questionnaire was done by the principal investigator. Data was entered using Epi-info and exported to SPSS for further cleaning and analysis purpose. Co-linearity diagnostic test was conducted to check for co-linearity between independent variables and the highest co-linearity; tolerance = 0.470 and VIF = 2.13 was found between latrine use and maternal history of diarrhea. Both bi-variable and multivariable analyses were used to compute the statistical associations. Variables in the bivariable analysis having a p-value < 0.2 were considered for multivariable analysis to adjust the confounders. The Hosmer–Lemeshow goodness of fit test (p = 0.700) was used to assess the fitness of the model. Statistical significances were declared at 95% CI and p-value < 0.05.

### Result

Out of 652 (326 model and 326 non-model) families, 322 (98.8%) model and 321 (98.5%) non-model families were enrolled during the data collection, with a total response rate of 98.6%.

#### Socio-demographic and economic characteristics of model and non-model families

All the respondents were real mothers in both groups. Three hundred ten (96.3%) model and 307 (95.6%) non-model families were rural residents. The age category for the majority of respondents, 178 (55.3%) of the model and 164 (48.0%) non-model families were ranged between 25 and 34 years (Table 1).

**Table 1:** Socio-demographic and economic characteristics of model and non-model families in L/Maychew district, Tigray, Ethiopia 2017

Variables	Categories	Model families		Non model families	
		Diarrhea		Diarrhea	
		Yes	No	Yes	No
		N (%)	N (%)	N (%)	N (%)
Relation of the child to the caretaker	Mother	26 (100)	296 (100)	65 (100)	256 (100)
	Care taker	0 (0)	0 (0)	0 (0)	0 (0)
Ethnicity	Tigray	26 (100)	296 (100)	65 (100)	256 (100)
	Other	0 (0)	0 (0)	0 (0)	0 (0)
Religion	Orthodox	26 (100)	296 (100)	65 (100)	256 (100)
	Other	0 (0)	0 (0)	0 (0)	0 (0)
Age of the mother/care taker in year	15–24	4 (15.4)	37 (12.5)	38 (14.8)	13 (20.0)
	25–34	17 (65.4)	161 (54.4)	135 (52.7)	29 (44.6)
	35–49	5 (19.2)	98 (33.1)	83 (32.4)	23 (35.4)
Educational level of the mother	No education	8 (30.8)	89 (30.1)	21 (32.3)	78 (30.5)
	1–4 grade	7 (26.9)	48 (16.2)	14 (21.5)	37 (14.5)
	5–8 grade	7 (26.9)	59 (19.9)	8 (12.3)	57 (22.3)
	9–12 grade	4 (15.4)	100 (33.8)	22 (33.8)	84 (32.8)
Marital status	Single	1 (3.8)	5 (1.7)	2 (3.1)	5 (2.0)
	Married	25 (96.2)	301 (98.3)	68 (97.8)	251 (98.1)
Occupation of the care taker	Farmer	25 (96.2)	278 (93.9)	62 (95.4)	241 (94.1)
	Other	1 (3.8)	18 (6.4)	3 (4.6)	15 (5.8)
Residence	Urban	0 (0)	12 (4.1)	3 (4.6)	11 (4.3)
	Rural	26 (100.0)	284 (95.9)	62 (95.4)	245 (95.7)
Sex of the selected child	Male	15 (57.7)	148 (50.0)	30 (46.2)	127 (49.6)
	Female	11 (42.3)	148 (50.0)	35 (53.8)	129 (50.4)
Age of the selected child in month	< 11	8 (30.8)	106 (35.8)	28 (43.1)	106 (41.4)
	12–35	16 (61.5)	132 (44.6)	27 (41.5)	108 (42.2)
	≥ 36	2 (7.7)	58 (19.6)	10 (15.4)	42 (16.4)
Wealth quintile	Poorest	8 (30.8)	70 (23.6)	9 (13.8)	47 (18.4)
	Poor	7 (26.9)	53 (17.9)	11 (16.9)	27 (10.5)
	Medium	3 (11.5)	57 (19.3)	14 (21.5)	52 (20.3)
	Wealthy	5 (19.2)	59 (19.9)	16 (24.6)	68 (26.6)
	Wealthiest	3 (11.5)	57 (19.3)	15 (23.1)	62 (24.2)

#### Hygiene, sanitation and behavioural characteristics

Having proper waste disposal method was more common among model families, 269 (83.5%) than non-model families, 172 (53.6%). Nearly 60% of both model and non-model families had handwashing facility. More than 80% of both model and non-model families use latrine for defecation. Two hundred eighty-four (88.2%) model families and 95 (30%) non-model families dispose their children’s feces into latrine. More than three-fourth of the model and non-model families had vaccinated their children both for Measles and Rotavirus vaccines (Table 2).

**Table 2:** Hygiene, sanitation and behavioural characteristics of model and non-model families in L/Maychew district, Tigray, Ethiopia 2017

Variables	Categories	Model HH		Non model HH	
		Diarrhea		Diarrhea	
		Yes	No	Yes	No
		N (%)	N (%)	N (%)	N (%)
Proper waste disposal site	Yes	13 (50.0)	256 (86.5)	21 (48.8)	151 (59.0)
	No	13 (50.0)	40 (13.5)	44 (64.7)	105 (41.0)
Feces seen around home	Yes	1 (3.8)	28 (9.8)	13 (20.0)	80 (31.2)
	No	25 (96.2)	268 (90.5)	52 (80.0)	176 (68.8)
Separate living area for domestic animals	Yes	7 (26.9)	101 (34.1)	27 (41.5)	117 (45.7)
	No	19 (73.1)	195 (65.9)	38 (58.5)	139 (54.3)
Water source	Tap	20 (76.9)	231 (78.0)	50 (76.9)	190 (74.2)
	Other source	6 (23.1)	65 (22.0)	15 (23.1)	66 (25.8)
Time take to fetch water (round trip) in minute	< 15	19 (73.1)	142 (48.0)	36 (55.4)	126 (49.2)
	16–30	5 (19.2)	133 (44.9)	26 (40.0)	116 (45.3)
	> 30	2 (7.7)	21 (7.1)	3 (4.6)	14 (5.5)
Water container have cover	Yes	21 (80.8)	233 (78.7)	44 (67.7)	194 (75.8)
	No	5 (19.2)	63 (21.3)	21 (32.3)	62 (24.2)
Hand washing facility	Yes	15 (57.7)	173 (58.4)	40 (61.5)	149 (58.2)
	No	11 (42.3)	123 (41.6)	25 (38.5)	107 (41.8)
Use latrine for defecation	Yes	10 (38.5)	249 (84.1)	46 (70.8)	213 (83.2)
	No	16 (61.5)	47 (15.9)	19 (29.2)	43 (16.8)
Child feces disposal method	Latrine	17 (65.4)	267 (90.2)	20 (30.8)	76 (29.7)
	Open disposal	9 (34.6)	29 (9.8)	45 (69.2)	180 (70.3)
Hands wash at a critical period	Yes	16 (61.5)	268 (90.5)	35 (53.8)	178 (69.5)
	No	10 (38.5)	28 (9.5)	30 (46.2)	78 (30.5)
Treat drinking water	Yes	24 (92.3)	234 (79.1)	51 (78.5)	211 (82.4)
	No	2 (7.7)	62 (20.9)	14 (21.5)	45 (17.6)
Duration of breast feed in month	≤ 12	6 (23.1)	30 (10.1)	8 (12.3)	40 (15.6)
	13–18	3 (11.5)	44 (14.9)	9 (13.8)	41 (16.0)
	19–24	15 (57.7)	194 (65.5)	46 (70.8)	149 (58.2)
	≥ 23	2 (67.7)	28 (9.5)	3 (3.1)	26 (10.2)
Measles vaccinated	Yes	22 (84.6)	250 (84.5)	55 (84.6)	216 (84.4)
	No	4 (15.4)	46 (15.5)	10 (15.4)	40 (15.6)
Rota virus vaccine vaccinated	Yes	22 (84.6)	237 (80.1)	49 (75.4)	192 (75.0)
	No	4 (15.4)	59 (19.9)	16 (24.6)	64 (25.0)

#### Diarrhea prevalence

Diarrheal disease in under five children was more common among non-model model families compared to model families. The occurrence of diarrheal disease in under-five children was 8.1% among model and 20.2% among non-model families with 95% CI 0.117, 0.168. Maternal history of diarrhea in the last 2 weeks preceding the survey was 49 (15.2%) among model and 67 (20.9) in non-model families.

#### Factors associated with diarrhea in under-five children

In the multivariable analysis; being non-model family, improper waste disposal method, having a maternal history of diarrhea and no latrine use during defecation had shown significant association with diarrhea in under-five children.

The occurrence of diarrhea in under-five children was 1.9 times more likely to happen among non-model families than model families (AOR = 1.9 and 95% CI 1.004, 3.565).

The odds of diarrhea in under-five children was 3.3 times higher among mothers who had diarrhea in the 2 weeks preceding the survey compared to those who had not (AOR = 3.3 and 95% CI 1.975, 5.570).

Diarrhea in under-five children was 2.6 times more likely to occur among families who practice improper waste disposal method compared to families who apply proper waste disposal method (AOR = 2.6 and 95% CI 1.251, 5.578).

Families who did not use latrine for defecation had two times higher odds of being diseased for diarrhea in under-five children than families use latrine (AOR = 2.1 and 95% CI 1.128, 3.897) (Table 3).

**Table 3:** Factors associated with diarrhoea in under-five children among model and non-model families in L/Maychew district, Tigray, Ethiopia 2017

Variables	Under-five children diarrhea		COR (95% CI)	AOR (95% CI)
	Yes	No		
	Number (%)	Number (%)		
Family type				
Model HH	26 (8.1)	296 (91.9)	Ref	
Non model HH	65 (20.2)	256 (79.8)	2.9 (1.780, 4.693)	1.9 (1.004, 3.565)*
Disposal method of child feces				
Dispose to latrine	37 (9.7)	343 (90.3)	Ref	
Open disposal	54 (20.5)	209 (79.5)	2.4 (1.524, 3.765)	1.5 (0. 823, 2.538)
Have proper waste disposal method				
Yes	34 (7.7)	407 (92.3)	Ref	
No	57 (28.2)	145 (71.8)	4.7 (2.956, 7.492)	2.6 (1.251, 5.578)*
Maternal history of diarrhea				
Yes	35 (30.2)	81 (69.8)	3.6 (2.241, 5.895)	3.3 (1.975, 5.570)*
No	56 (10.6)	471 (89.4)	Ref	
Hand wash at critical period				
Yes	51 (10.3)	446 (89.7)	Ref	
No	40 (27.4)	106 (72.6)	3.3 (2.073, 5.254)	0.9 (0.423, 1.773)
Latrine use				
Yes	56 (10.8)	462 (89.2)	Ref	
No	35 (38.5)	90 (72.0)	3.2 (1.987, 5.179)	2.1 (1.128, 3.897)*

*COR* crude odds ratio, *AOR* adjusted odds ratio and *significant at p-value ≤0.05

### Discussion

There was a significant difference on the prevalence of diarrhea in under-five children among model and non-mode families. The prevalence of diarrhea among model and non-model families was 8.1% and 20.2% respectively with 95% CI 0.117, 0.168. The difference might be due to the application of the health extension’s training and demonstrations on personal hygiene, sanitation, and water safety measures by the model families while non-model families did not This was consistent with the findings from Hawassa and Shenko, south-west Ethiopia in which the prevalence of under-five diarrhea was higher among non-model families than model families [4, 13].

The odd of diarrhea among under-five children was two times higher among health extension non-model families than model families. This is consistent with findings in Hawasa and Shenko district, Ethiopia [4, 13]. This might be due to the reason that the health extension workers deliver training, support and follow up on a package of basic and essential preventive and curative health services targeting at HHs in a community to those who were intended to be models for others. If the health knowledge and skill are appropriately transferred, model families may take responsibility for producing and maintaining their children’s health. Then this training, support, and follow up might made them practice health extension packages well compared to non-model families.

The odds of diarrhea in under-five children was found higher among children with mothers who had diarrhea in the 2 weeks period preceding the survey compared to those who had not. The reason could be due to the characteristics of diarrhea that can be transmitted from a mother with diarrheal disease to her child through contaminated vehicles like water and food due to poor personal hygiene.

Diarrhea in under-five children was more likely to occur among families who practice improper waste disposal method compared to families who apply proper waste disposal method. Similarly, finding was reported in Shenko district South West Ethiopia [13]. Unsanitary environment allows diarrhea-causing pathogens to spread more easily [8]. It is known that if wastes are disposed of improperly, children’s may be easily accessed and have direct contact with the wastes which are the means of diarrhea transmission.

The probability of under-five diarrhea was higher among families who do not use latrine than families who use latrine. This is in line with the studies from Dejen district and Benishangul Gumuz, Ethiopia [6, 14]. Latrine availability decreases feco-oral contamination in the domestic environment and, in turn, this prevents spread of disease-causing organisms to human beings [4]. Most pathogens that cause diarrhea’s mode of transmission is feco-oral [8]. If families have no habit of latrine utilization, children’s may have the possibility of contacting with diarrheal pathogens where there are feces in the environment.

### Conclusion

The prevalence of diarrhea in under-five children among non-model families was higher than model families. Being non-model family, maternal history of diarrhea in the 2 weeks preceding the survey, waste disposal and latrine utilization were significantly associated factors with diarrhea in under-five children. The district health office in general and the health extension workers, in particular, need to focus on utilization of health extension packages so as to make the entire families model. Therefore health extension model families training, follow up, support and behavior change program need to be expanded for all non-model families. HEWs are expected to deliver health education for families on proper waste disposal method and latrine utilization. Furthermore, when they visit families, they have to teach mothers how to care their children and handle food and water safety during maternal diarrheal illness.

## Limitation of the study

There may be recall bias introduced into the study because the maternal history of diarrhea and diarrhea in under five children in 2 weeks period prior to the data collection time were allowed respondents to recall histories which are doubtful to remember exactly what happened in the past.

## Data Availability

Data and other required materials can be submitted if requested.

## Abbreviations

AIDS: acquired immune deficiency syndrome
CI: confidence interval
HH: household
HEP: health extension program
HSDP: Ethiopian Health Sector Development Program
HSEP: Health Service Extension Program
TB: tuberculosis
UNICEF: United Nations Children’s Fund
WHO: World Health Organization
